# How to Translate DNA Methylation Biomarkers Into Clinical Practice

**DOI:** 10.3389/fcell.2022.854797

**Published:** 2022-02-23

**Authors:** Wolfgang Wagner

**Affiliations:** ^1^ Helmholtz-Institute for Biomedical Engineering, Stem Cell Biology and Cellular Engineering, RWTH Aachen University Medical School, Aachen, Germany; ^2^ Center for Integrated Oncology Aachen Bonn Cologne Düsseldorf (CIO ABCD), Aachen, Germany

**Keywords:** epigenetic, biomarker, hallmarks, DNA methylation, aging, blood counts, methods, IVDD

## Abstract

Recent advances in sequencing technologies provide unprecedented opportunities for epigenetic biomarker development. Particularly the DNA methylation pattern—which is modified at specific sites in the genome during cellular differentiation, aging, and disease—holds high hopes for a wide variety of diagnostic applications. While many epigenetic biomarkers have been described, only very few of them have so far been successfully translated into clinical practice and almost exclusively in the field of oncology. This discrepancy might be attributed to the different demands of either publishing a new finding or establishing a standardized and approved diagnostic procedure. This is exemplified for epigenetic leukocyte counts and epigenetic age-predictions. To ease later clinical translation, the following hallmarks should already be taken into consideration when designing epigenetic biomarkers: 1) Identification of best genomic regions, 2) pre-analytical processing, 3) accuracy of DNA methylation measurements, 4) identification of confounding parameters, 5) accreditation as diagnostic procedure, 6) standardized data analysis, 7) turnaround time, and 8) costs and customer requirements. While the initial selection of relevant genomic regions is usually performed on genome wide DNA methylation profiles, it might be advantageous to subsequently establish targeted assays that focus on specific genomic regions. Development of an epigenetic biomarker for clinical application is a long and cumbersome process that is only initiated with the identification of an epigenetic signature.

## Introduction

Epigenetics is a fascinating branch of research that will increasingly find its way into clinical diagnostics. There are different types of epigenetic modifications, including DNA methylation (DNAm), histone modifications, and higher order chromatin conformation (Zhu et al., 2013). For epigenetic diagnostics DNAm appears to be best suited, as it can be precisely determined at specific cytosine residues of CG dinucleotides (CpG sites) (Smith and Meissner, 2013). The enzymes that catalyze *de novo* methylation and maintenance of DNAm levels have long been known (Okano et al., 1999; Reik et al., 2001). In contrast, despite the impressive scientific descriptions of the epigenetic landscape in the last years, it remains unclear how these enzymes are directed to specific sites in the genome and how the complex epigenetic network is orchestrated. In the future, a better understanding of the underlying regulatory mechanisms that direct DNAm can help to derive even more focused and functionally relevant biomarkers. Nonetheless, the currently rather descriptive analysis of DNAm alterations can already provide accurate insights into cellular and developmental processes and thus provide valuable diagnostic tools.

## Fields of Application for DNA Methylation Biomarkers

An epigenetic biomarker can be defined as any epigenetic mark, which is stable and reproducible during sample processing and can be measured in body fluids or primary tissue. They provide tools for diagnosis, prognosis, monitoring of disease evolution, and can support clinical decision-making (Garcia-Gimenez et al., 2017). The scope of DNAm biomarkers can be classified in four categories:

1) Cellular composition. Since DNAm is fundamentally linked to cellular development, it is very well suited to provide insights into cellular composition of a given tissue. Each of our cell types has a characteristic DNAm pattern (Moss et al., 2018). Tissue composition can then be reliably estimated using deconvolution algorithms, as each cell only has two copies of chromosomal DNA (Houseman et al., 2012; Schmidt et al., 2020). With such assays it is for instance possible to investigate the origin of cell-free DNA in blood plasma as a liquid biopsy of potentially affected tissues (Moss et al., 2018; Neuberger et al., 2022).

2) Environmental influences and lifestyle. For example, cigarette smoking affects the DNAm levels at multiple genomic loci (Tsaprouni et al., 2014) and this effect can be reliably tracked in blood and brain specimen (Gadd et al., 2021). There are also DNAm biomarkers for alcohol consumption (Liu et al., 2018) and other parameters, such as physical activity, exercise and body weight, which can impact on the epigenome.

3) Aging. It is remarkable how well DNAm correlates at specific CpGs with chronological age (Horvath, 2013; Weidner and Wagner, 2014). Corresponding epigenetic signatures, so-called “epigenetic clocks”, can support forensic investigation of blood traces or of donors with allegedly unknown age. On the other hand, there is increasing evidence that epigenetic age more closely reflects biological age than chronological age (Bell et al., 2019). In fact, accelerated epigenetic age is associated with higher all-cause mortality in later life (Marioni et al., 2015). Whether pure epigenetic clocks can be trained to better capture aspects of biological age—independent from epigenetic changes by other confounding parameters of life-style, cellular composition, and diseases—still requires further validation. Either way, a biomarker for biological age has great potential for individualized medicine to evaluate therapeutic options. In addition, such studies can help to uncover factors that influence aging to adjust life-style for healthy aging.

4) Diseases. So far, epigenetic diagnostic biomarkers have almost exclusively been established for applications in oncology (Locke et al., 2019). Epigenetic aberrations can mimic genomic mutations, e.g., by DNAm aberrations in the gene *DNMT3A* in acute myeloid leukemia (Jost et al., 2014). In fact, next to driver mutations it seems to be particularly epigenetic aberrations that cause malignant transformation (Schoofs et al., 2014; Wagner et al., 2015), and such epimutations may either arise in the absence of DNA sequence changes (primary epimutations), or secondary to genetic variants (secondary epimutation) (Cerrato et al., 2020). In contrast, episignatures rather resemble complex aberrant DNAm patterns that are not functionally restricted to specific sites in the genome (Bozic et al., 2022). Epimutations as well as episignatures can provide insight into the malignant clone and can be used for initial diagnostics and disease stratification. Furthermore, DNAm patterns can be used to predict response to a specific therapeutic regimen and to track measurable residual disease after treatment (Bozic et al., 2022). Although there are currently no approved *in vitro* diagnostic (IVD) tests targeting methylation outside of oncology, there is clear evidence that many other diseases, including imprinting disorders, neurodegenerative and psychiatric disorders, involve epigenetic aberrations that may be addressed by epigenetic signatures (Beltran-Garcia et al., 2019; Taryma-Lesniak et al., 2020).

In view of the rapid development in DNAm studies on the one hand, and the increasing regulatory requirements on the other hand, it is to be expected that the gap between potential applications and actual implementations will continue to widen. Two examples of potential applications are further highlighted below.

### Epigenetic Leukocyte Counts

Leukocyte counts in blood is one of the most common diagnostic tests, which is conventionally performed with automated cell counting devices and particularly for stratification of lymphocyte subsets with flow cytometry (Pitoiset et al., 2018). Yet, deconvolution of leukocyte subsets based on DNAm may have several advantages as compared to the conventional regimen (Sontag et al., 2022): 1) It is applicable to very small volumes of blood that can be harvested by a finger-prick; 2) DNAm analysis is possible with frozen blood; 3) it might be applied to coagulated samples or specimen with ineffective antibody binding; and 4) the precise measurement of DNAm levels might provide less variability in inter-laboratory comparison. Deconvolution models were initially developed based on cell-type specific hypo- or hypermethylation that was analyzed in purified leukocyte subsets (Houseman et al., 2012). Derivation of such signatures for granulocytes, lymphocytes, B cells, T cells (CD4 or CD8), NK cells, and monocytes was particularly based on Infinium BeadChip mircoarrays (Accomando et al., 2014; Salas et al., 2018).

It has been demonstrated that leukocyte counts can also be determined by targeted assays at specific CpGs. These assays are based on quantitative PCR (qPCR), pyrosequencing or digital droplet PCR (ddPCR) (Baron et al., 2018; Frobel et al., 2018; Malic et al., 2019) and appear to be applicable for clinical use. We have recently further optimized and validated our targeted pyrosequencing and ddPCR assays for leukocyte deconvolution using 332 venous and 122 capillary blood samples from healthy donors (Sontag et al., 2022). In addition, we tested 36 samples from ring trials and venous blood from 266 patients diagnosed with different hematological diseases. Overall, the targeted DNAm analysis by pyrosequencing or ddPCR is a valid alternative to quantify leukocyte subsets (Sontag et al., 2022). However, much research remains necessary for further optimization, validation, and approval as an *in vitro* diagnostic device (IVDD) before epigenetic blood counts can ultimately find their way into clinical application.

### Epigenetic Clocks

As already mentioned above, a multitude of epigenetic signatures have been described to estimate either chronological age or biological age. Ten years ago, our group was one of the first to describe such epigenetic clocks (Bocklandt et al., 2011; Koch and Wagner, 2011). Since then, these signatures significantly improved by the rapidly growing number of available Infinium BeadChip datasets and more elegant bioinformatic considerations (Horvath, 2013). Integration of Infinium BeadChip measurements facilitated human biomarker development, that was unparalleled in other species (Wagner, 2017). Only recently, the Infinium Mouse Methylation BeadChip became available, as well as the mammalian methylation array that covers highly conserved CpGs (Arenson et al., 2022).

In the future, epigenetic aging signatures might move from measuring DNAm levels at individual CpGs to a probabilistic analysis of the binary sequel of methylated and non-methylated CpGs on individual reads. This method was first described for barcoded amplicon sequencing and may reflect heterogeneity of epigenetic aging within a sample (Han et al., 2020a; Han et al., 2020b). It was further developed within the scAge framework that is applicable with few bisulfite sequencing reads (Trapp et al., 2021). Probabilistic epigenetic age-predictions could be performed with tagmentation-based indexing for methylation sequencing (TIME-Seq) that is applicable for Methyl-ATAC-seq with low-cost shallow sequencing (Griffin et al., 2021). It might eventually even be used for single-cell methyl-ATAC-seq data to ultimately elucidate heterogeneity of epigenetic aging within a given sample. Other sequencing technologies with longer reads, such as nanopore sequencing or PacBio sequencing, will further strengthen such probabilistic pattern-based approaches. While the shallow-sequencing may significantly reduce the sequencing costs, the bioinformatic demands are relatively high and further validation is still elusive. So far, these methods can hardly be standardized to be accredited as diagnostic procedure (e.g., according to ISO13485).

For a clinical or forensic application of epigenetic clocks, it might be advantageous to rather focus on a few individual age-associated CpGs with targeted methods (Weidner et al., 2014). In fact, some individual CpGs reveal very high correlation with chronological age and they also seem to capture aspects of biological aging (Lin et al., 2016). Targeted epigenetic clocks for pyrosequencing, MassArray, ddPCR, or barcoded amplicon sequencing can reach almost similar precision as described for Infinium BeadChip clocks (Han et al., 2020a). They provide a tradeoff between the less CpGs to be integrated into a robust network for epigenetic age-predictions, and the more accurate DNAm measurements at individual CpGs. Lastly, it is worth mentioning that aging is *per se* not a disease and therefore, depending on the application, an accreditation for clinical use may not be necessary.

## Hallmarks for DNA Methylation Biomarkers

**FIGURE 1:**
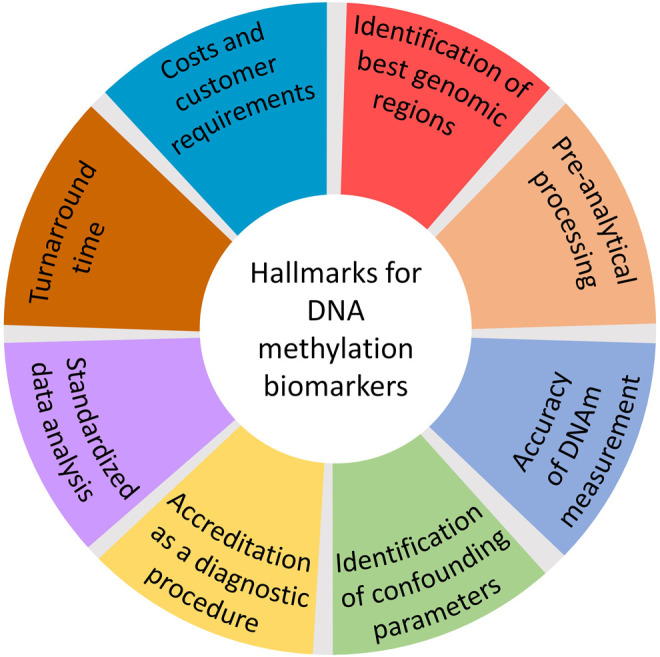
Aspects to be considered when designing diagnostic epigenetic biomarkers.

To successfully establish an IVDD it is important to benchmark accuracy and robustness in comparison to conventional markers. The sensitivity (ability of the test to correctly identify patients with a disease) and specificity (ability to correctly identify a subject without the disease) are dependent on a multitude of variables in the analytical process (Taryma-Lesniak et al., 2020). Many methods for DNAm analysis have evolved and can be classified in genome-wide approaches or targeted measurements at specific genomic regions (Locke et al., 2019) (Table 1). Each of these methods has advantages and disadvantages that can only be briefly touched in the context of this mini-review. It is not possible to give general applicable guidelines for development of epigenetic biomarkers due to the very different fields of application, molecular biological features, and clinical requirements. However, the following parameters should be considered when designing such studies (Figure 1).

**TABLE 1:** Comparison of selected methods for DNAm analysis.

Method	Mechanism for DNAm detection	Genome wide analysis	Targeted DNAm analysis	Accuracy of DNAm at individual CpGs	Integrative analysis with other datasets	Scalability for many samples	CE accredited instrumentation ^a^	Bioinformatic requirement	Usual turn arround time	Costs ^b^
Whole Genome Bisulfite Sequencing (WGBS)	High-throughput sequencing of bisulfite or enzym. converted DNA	++	—	+	++	-	+	↑↑	↑↑	↑↑
Reduced Representation Bisulfite Sequencing (RRBS)	High-throughput sequencing of bisulfite or enzym. converted DNA	+	—	+	+	+	—	↑↑	↑↑	↑
Infinium BeadChip Technology (EPIC array)	Microarray analysis of bisulfite-converted DNA	+	—	++	++	+	—	↑	↑	↑
Nanopore sequencing	Detection of current changes during sequencing of non-converted DNA	++	—	-	+	+	+	↑↑	↑	↑
Methylated DNA immunoprecipitation (MeDIP)	Affinity capture of unconverted DNA with antibody and deep sequencing	++	NA	-	+	-	—	↑↑	↑↑	↑
Methyl Binding Domain (MBD) Sequencing	Affinity capture of unconverted DNA with MBD and deep sequencing	++	NA	-	+	-	—	↑↑	↑↑	↑
Probabilistic frameworks for genome wide DNAm pattern analysis	Shallow sequencing data (e.g. RRBS for scAge or methyl-ATAC-seq for TIME-seq)	-		-	+	++	—	↑↑	↑↑	↔
Methylation sensitive restriction enzyme (MSRE) PCR	Unconverted DNA is digested with MRSE and amplified by PCR	NA	+	-	-	+	—	-	↔	↓
Methylation specific PCR	PCR amplicons of bisulfite-converted or enzymatic converted DNA	NA	+	-	-	+	—	-	↔	↓
Quantitative PCR (qPCR)	Allele specific qPCR after bisulfite-conversion	NA	+	+	-	+	—	-	↔	↓
Targeted amplicon sequencing	Deep-sequencing of PCR amplicons of bisulfite converted DNA	NA	+	++	-	++	+	↑	↑	↔
Pyrosequencing	Sequencing of PCR amplicons of bisulfite-converted DNA	NA	+	++	-	+	—	-	↔	↔
Digital droplet PCR (ddPCR)	PCR of bisulfite-converted DNA in droplets	NA	+	++	-	+	+	-	↔	↔
EpiTYPER	Mass-spectrometry of bisulfite-converted DNA	NA	+	++	-	+	—	-	↔	↔

This table shall only exemplify how hallmarks of DNAm biomarkers may be influenced by different methods. It does not claim to represent all available approaches for DNAm analysis. The suggested classifications will vary between applications and laboratories.

a Accreditation may also include enzymes and viable components. The regulatory requirements and accreditation may change with time and according to local regulations.

b The expenses can only be estimated relatively. They include consumables, instrumentation and personnel costs and depend largely on the number of samples that can be processed in parallel (and on the number of CpGs for the targeted assays). NA, not applicable.

### Identification of the Best Genomic Regions

There are approximately 28 million CpG sites in the human genome and selection of the best suited regions is the first challenge for biomarker development. Whole Genome Bisulfite Sequencing (WGBS) provides insight into the entire methylome based on unbiased genome wide DNAm analysis, but this necessitates high coverage of deep-sequencing reads, which is relatively costly. Reduced Representation Bisulfite Sequencing (RRBS) can significantly reduce the sequencing effort, but coverage at specific locations of the genome sometimes deviates significantly, reducing transferability. Within the last decade human biomarker development has been revolutionized by Infinium BeadChip technology. These microarray platforms covered initially approximately 27,000 CpGs (27k), later 450,000 CpGs (450k), and currently, with the human EPIC BeadChip, approximately 850,000 CpGs (Pidsley et al., 2016). A major advantage of this method is that many DNA methylation profiles of public databases can be directly compared and integrated into the analysis to identify and validate suitable signatures. For any epigenetic biomarker development, it is crucial to have a high number of measurements for a training set and for an independent validation cohort.

### Pre-Analytical Processing

Sample harvesting, transportation, DNA extraction, treatments such as bisulfite conversion or enzymatic conversion, storage and preservation can have enormous impact on the final results. Since biomarker development requires large training and validation cohorts, biobanks play a crucial role for the initial development of personalized medicine (Peiró-Chova et al., 2016). However, sample processing may be very different in biobanks as compared to a later clinical setting. The samples may even be formalin-fixed paraffin-embedded (FFPE) tissue specimens, which severely affects fragmentation and down-stream analysis of the DNA. It is important to consider the best clinical procedure for sample collection when designing an epigenetic biomarker. For example, blood samples might be taken from venous blood or capillary blood by finger pricks, shipped as dried blood spot or at liquid state, frozen or at room temperature (Sontag et al., 2022). For liquid biopsies, it may also be relevant if DNA is extracted from serum or plasma (Constancio et al., 2020), or if the donor performed physical activity before sample collection (Neuberger et al., 2022). Similar considerations are also required for specimen from urine, stool, airway, or other tissues (Locke et al., 2019). There are many alternative protocols for DNA extraction and conversion that may need to be accredited for the IVDD, too. Depending on the number of samples, barcoding and automation need to be considered.

### Accuracy of DNA Methylation Measurement

In contrast to other epigenetic modifications, such as the histone code, the DNAm level can be determined precisely for each CpG, as absolute value ranging from 0 to 100%. For many biomarkers the difference of DNAm levels is relatively low and therefore the accuracy of DNAm measurement is an important parameter. This applies particularly for epigenetic biomarkers that do not simply classify between two DNAm states (yes or no), but rather resemble a continuous variable (as for example in epigenetic leukocyte counts and epigenetic clocks). In this case small deviations in the accuracy of DNAm measurements can have big impact on predictions, even though the biological relevance and the phenotype effect of small changes in DNAm is still under debate. For the deep-sequencing based approaches accuracy depends largely on the sequencing depth. In contrast, Infinium BeadChip microarrays achieve overall relatively high precision, while there is batch-to-batch and inter-laboratory variation, which can be partially compensated for by background correction and normalization procedures (Pidsley et al., 2016). Even higher accuracy and precision of DNAm levels at specific CpGs can be achieved by targeted measurement techniques (Blueprint-consortium, 2016). For instance, by pyrosequencing, mass-spectrometry based EpiTYPER, or bisulfite amplicon sequencing (BA-seq) DNAm differences below 5% are detectable. However, these targeted methods can have an inherent PCR bias if either methylated or non-methylated sequences are preferentially amplified. A possible exception is digital droplet PCR (ddPCR): The bisulfite-converted DNA is dispersed into small droplets with individual PCR reactions, which are either detected as positive or negative for methylated and non-methylated sequences. The exact DNAm values can then be calculated using Poisson distribution for the different droplets. In fact, there is some evidence that DNAm measurement with ddPCR has even higher accuracy than pyrosequencing, but this may largely depend on the assay design (Han et al., 2020a; Sontag et al., 2022). Either way, inter-laboratory comparison and round robin tests should be considered.

### Identification of Confounding Parameters

For clinical applications it is necessary to have a good understanding of confounding factors. Environmental and life-style parameters, aging, cellular composition, and diseases can affect the signatures (Gadd et al., 2021). Furthermore, there are DNAm differences between woman and men, particularly at sex chromosomes. For example, epigenetic age predictions of women are overall under-estimated as compared to men, which might be attributed to their longer life expectancy (Horvath, 2013; Weidner et al., 2014). In addition, epigenetic characteristics can differ between ethnic groups (Becker et al., 2021). Such confounding parameters can be identified by epigenome-wide association studies (EWAS), that have enormous power when utilizing large available datasets. It is important to envisage potential confounding parameters already at study design but they can ultimately only be identified through extensive studies with many patient samples in the validation phase.

### Accreditation as a Diagnostic Procedure

An approved diagnostic test needs to be applicable reliably and standardized for many years. For clinical application diagnostic tests are required to have a quality certificate, for example a CE mark from the European Union as an *in vitro* diagnostic device (IVDD) according to IVDR2017/746, which will be mandatory to be accomplished by all diagnostic devices in May 2022, or approval from the Food and Drug Administration (FDA) (Locke et al., 2019). The process to get such approvals is based on validation and benchmarking experiments — it takes years and is very cost-intensive. Also, all instruments need to have certificates for clinical use, which is not always the case. Complex analytical procedures with many suppliers tend to be detrimental to clinical implementation. For example, the changes in Infinium BeadChip platforms (from 27k, to 450k, to EPIC) and their continuous annotation updates are not a major obstacle for basic research, but they are a challenge for an already accredited process. With this in mind, it may be advantageous to keep diagnostic procedures as simple as possible and to rather rely on targeted assays for a higher and consistent throughput.

### Standardization of Data Analysis

For an IVDD all processes must be fully standardized, including data analysis. For epigenetic studies the bottleneck is often bioinformatic evaluation. Not only it is becoming increasingly difficult to recruit specially trained bioinformaticians, there are countless integration possibilities of different datasets and the rapidly growing arsenal of tools can hardly be overseen, even by experts. Furthermore, standardized collection of DNAm data and clinical information is critical. Every program that is used needs to be completely described and approved, even for simple linear regression equations. The programs used for analysis of sequencing data must also be accredited accordingly. Thus, it is also important to simplify and standardize the data analysis procedures.

### Turnaround Time

The turnaround time can be crucial for clinical application. The procedures usually comprise DNA isolation and bisulfite conversion, which already require amost one day. Targeted methods, such as pyrosequencing, ddPCR, or EpiTYPER necessitate only a few additional PCR and/or sequencing steps and the results may therefore be available after one or two days (Blueprint-consortium, 2016). In contrast, genome-wide methods and amplicon sequencing are often subject to longer waiting times due to the high frequentation of sequencing instruments. Quality control and bioinformatic evaluation of deep-sequencing data may also require several additional days. For the final decision on the method of the diagnostic procedure, the time aspects should be taken into account — not least because personnel costs represent a significant component of the process costs.

### Costs and Customer Requirements

It is advantageous to consider potential customers and marketing already at the time of the initial study design. How would the assay compare with conventional assays? Would it rather be distributed as kit or service? For example, customers in forensics may prefer in-house analysis—in this case a kit for commonly available instrumentation might be advantageous. On the other hand, DNA is relatively stable and can be shipped at room temperature (in contrast to RNA, which is bound to rapid degradation). The samples can be frozen for many years prior to measurement, making retrospective studies possible. Therefore, the samples might alternatively be easily shipped to a service provider for a centralized analysis. Due to the personnel costs the price per assay may largely depend on the number of samples that can be processed in parallel. Automation and parallelization of sample acceptance, DNA isolation, bisulfite conversion (or enzymatic conversion), amplification and measurement, up to the evaluation and the generation of a final report is therefore to be aimed for.

## Conclusion

There is a “valley of death” between identification of promising epigenetic signatures and translation into clinical practice. Despite the enormous diagnostic potential and rapidly growing numbers of described epigenetic signatures only a small number of epigenetic biomarkers are approved as IVDD (Beltran-Garcia et al., 2019; Locke et al., 2019; Taryma-Lesniak et al., 2020). To bridge this gap, it is necessary to already focus on the clinical demands of a potential epigenetic biomarker at the initial study design. While the genomic regions are usually identified with genome-wide approaches, clinical application may require further translation into a robust and highly standardized targeted assay that rather uses a small number of CpGs. The scientific community needs to acknowledge that optimization, validation, and standardization of existing epigenetic signatures is important research to establish diagnostic DNAm biomarkers.
